# Associations of Human Colorectal Adenoma with Serum Biomarkers of Body Iron Stores, Inflammation and Antioxidant Protein Thiols

**DOI:** 10.3390/antiox10081195

**Published:** 2021-07-27

**Authors:** Ben Schöttker, Xīn Gào, Eugène HJM Jansen, Hermann Brenner

**Affiliations:** 1 Division of Clinical Epidemiology and Aging Research, German Cancer Research Center, 69120 Heidelberg, Germany; xin.gao@dkfz.de (X.G.); h.brenner@dkfz.de (H.B.); 2 Network Aging Research, Heidelberg University, 69115 Heidelberg, Germany; 3 Centre for Health Protection, National Institute of Public Health and the Environment, 3721 MA Bilthoven, The Netherlands; eugenejansen99@gmail.com; 4 Division of Preventive Oncology, German Cancer Research Center and National Center for Tumor Diseases (NCT), 69120 Heidelberg, Germany; 5 German Cancer Consortium (DKTK), German Cancer Research Center, 69120 Heidelberg, Germany

**Keywords:** thiols, C-reactive protein, iron, ferritin, transferrin saturation, colorectal adenoma

## Abstract

Red and processed meat consumption and obesity are established risk factors for colorectal adenoma (CRA). Adverse changes in biomarkers of body iron stores (total serum iron, ferritin, transferrin and transferrin saturation), inflammation (high-sensitivity C-reactive protein [hs-CRP]) and anti-oxidative capacity (total of thiol groups (-S-H) of proteins [SHP]) might reflect underlying mechanisms that could explain the association of red/processed meat consumption and obesity with CRA. Overall, 100 CRA cases (including 71 advanced cases) and 100 CRA-free controls were frequency-matched on age and sex and were selected from a colonoscopy screening cohort. Odds ratios (OR) and 95% confidence intervals (95%CI) for comparisons of top and bottom biomarker tertiles were derived from multivariable logistic regression models. Ferritin levels were significantly positively associated with red/processed meat consumption and hs-CRP levels with obesity. SHP levels were significantly inversely associated with obesity. Transferrin saturation was strongly positively associated with overall and advanced CRA (ORs [95%CIs]: 3.05 [1.30–7.19] and 2.71 [1.03–7.13], respectively). Due to the high correlation with transferrin saturation, results for total serum iron concentration were similar (but not statistically significant). Furthermore, SHP concentration was significantly inversely associated with advanced CRA (OR [95%CI]: 0.29 [0.10–0.84]) but not with overall CRA (OR [95%CI]: 0.65 [0.27–1.56]). Ferritin, transferrin, and hs-CRP levels were not associated with CRA. High transferrin saturation as a sign of iron overload and a low SHP concentration as a sign of redox imbalance in obese patients might reflect underlying mechanisms that could in part explain the associations of iron overload and obesity with CRA.

## 1. Introduction

Colorectal cancer (CRC) is a global public health problem. In 2020, CRC was the third most commonly diagnosed malignant neoplasm and the second most common cause of cancer deaths worldwide [1]. Colorectal adenoma (CRA) is the precursor of most CRCs and reducing the burden of risk factors for their development is the main aim of primary prevention efforts against CRC [2,3]. Common risk factors for CRA and CRC are smoking, excessive alcohol consumption, physical inactivity, obesity and high red or processed meat consumption [3,4]. These lifestyle factors are interrelated and especially obesity can result in conditions of systemic, subclinical inflammation and oxidative stress [5,6], which may be pathways towards CRC development [7,8]. 

Inflammation is commonly evaluated by measuring C-reactive protein (CRP), which is an acute-phase protein and is produced in response to inflammatory stimuli in hepatic tissue. Increased CRP levels not only indicate acute inflammation, but also are closely related to oxidative stress in pathological conditions, such as hypertension, dilated cardiomyopathy and ischemic stroke [9,10,11]. In clinical practice, the high-sensitivity CRP (hs-CRP) test, which is more sensitive than a standard test in the region of 0.2–5 mg/L, is widely used to evaluate the risk of developing cardiovascular disease [12]. However, little is known about the associations of hs-CRP levels with CRA. 

Oxidative stress refers to a disruption of redox signaling control pathways, which results from an imbalance of the anti-oxidants defense system and pro-oxidants, such as reactive oxygen species (ROS) [13]. Direct measurement of ROS in human specimens is rather difficult because of their short half-life. However, oxidative stress can be reflected by concentrations of ROS metabolites and anti-oxidant substances in blood or urine samples. The total of thiol groups (-S-H) of proteins and peptides [SHP] concentration is regarded as a stable biomarker for the overall antioxidant capacity because of the reducing property of the carbon-bonded sulfhydryl (R-SH) group of thiols [14]. In blood, the most frequent proteins and peptides with thiol groups include albumin, glutathione and members of the thioredoxin family [15]. A considerable body of evidence from biological and epidemiological studies supports the role of oxidative stress in the promotion of carcinogenesis [16,17,18,19]. However, studies on the associations of anti-oxidant capacity biomarkers and CRA are sparse.

Red meat has a high concentration of haem that can readily become nitrosylated and act as a nitrosating agent [20]. Many *N*-nitroso compounds are carcinogenic and, especially for CRC, evidence for a causal relationship is convincing [21]. Dietary haem is a source of the human body to fill its iron stores. Indicators of body iron stores include non-bound iron ions, ferritin (total iron stores), and transferrin (iron-binding capacity). In addition, transferrin saturation, which is calculated as the percentage of iron bound to transferrin, reflects the availability of circulating iron. Meta-analyses of observational studies reported that high dietary intake of haem iron is associated with CRC incidence [4,22]. Nevertheless, only a limited number of epidemiological studies investigated the association between serum iron markers and CRA, and their findings were inconclusive [22,23,24,25,26,27].

The aim of our investigation was to assess whether serum biomarkers of body iron stores (ferritin, transferrin, total serum iron and transferrin saturation), inflammation (hs-CRP) and anti-oxidant capacity (SHP) are associated with overall and advanced CRA in a colonoscopy screening cohort.

## 2. Materials and Methods

### 2.1. Study Design and Study Population

Our analysis is based on data from the ongoing BliTz study (German name: Begleitende Evaluierung innovativer Testverfahren zur Darmkrebsfrüherkennung), which was initiated in 2006 with the primary aim of evaluating non-invasive CRC screening tests by comparison with findings at screening colonoscopy. The design of the BliTz study has been reported in detail by previous publications [28,29,30]. In brief, participants of the German screening colonoscopy program are being recruited in cooperation with 20 gastroenterology practices in southern Germany. They are invited to provide blood and stool samples at a preparatory visit. Basic information on CRC risk and preventive factors, including age, sex, smoking status, physical activity, red and processed meat consumption are collected by using a standardized questionnaire at the preparatory visit. Findings at screening colonoscopy are abstracted from colonoscopy and histopathology records, and participants are classified according to the most advanced finding at colonoscopy. The study was approved by the Ethics committee of Heidelberg University, Germany and written informed consent was obtained from all participants (protocol code 178/2005 and date of approval 17 June 2005).

At the time of the sample picking for this project (May 2012), 4508 participants with blood and stool samples were included in the database of the BLITZ study. In a case-control design, 71 advanced and 39 non-advanced CRA cases were randomly selected and 100 participants without colorectal neoplasms, who were frequency-matched on age (±5 years) and sex to CRA cases were selected for biomarker measurements.

### 2.2. Definition of Key Variables

Information on red and processed meat consumption was based on reported consumption frequencies and combined into one variable with three categories. Low consumption was defined as both red and processed meat consumption no more than once per week. High consumption was defined as daily consumption of both red and processed meat or consumption of either red or processed meat consumption several times per day. All other participants were grouped in the intermediate meat consumption category. Body mass index (BMI) was calculated based on reported height and weight. CRA with at least one of the following features were defined as advanced CRA: size > 1 cm, tubulovillous or villous components, or high-grade dysplasia.

### 2.3. Laboratory Measurements

Blood samples were collected from an antecubital vein, serum was derived, aliquoted and stored at −80 °C until analysis. For measurements of selected biomarkers, one serum aliquot of 100 µL was shipped on dry ice to the Laboratory for Health Protection Research (Bilthoven, The Netherlands). All laboratory measurements were conducted blinded with respect to findings at screening colonoscopy. In this study, the coefficients of variation were 0.8%, 3.2%, 8.5%, 1.8% and 2.8% for SHP, hs-CRP, ferritin, transferrin and total serum iron concentration, respectively. The following assays were used by following manufacturers’ manuals and were adapted to an automatic analyzer (LX20-Pro, Beckman-Coulter Diagnostics, Woerden, The Netherlands).

#### 2.3.1. SHP

The kit to measure SHP was purchased from Rel Assay Diagnostics (Gaziantep, Turkey). The SHP test quantifies the presence of sulfhydryl (–SH) groups in the biological sample. When serum/plasma is used as a sample, the -SH groups are present mainly in proteins. The method is based on the capacity that the –SH groups react with 5,5′-dithiobis-2-nitrobenzoic acid, followed by a development of a colored complex that can be measured with an autoanalyzer at 405 nm absorbance. l-cysteine was used as standard. The range of detection is 0.1–1.8 mmol SH-groups in proteins. The reference range for SHP is 450–680 μmol/L. At a level of 660 μmol/L, the interassay coefficient of variation is 2.5% (*N* = 9).

#### 2.3.2. hs-CRP

The kits for hs-CRP were purchased from Beckman Coulter Diagnostics (Woerden, The Netherlands). The high-sensitive CRP assay is based on a highly sensitive particle immunoassay rate methodology. An anti-CRP antibody-coated particle binds to CRP in the patient sample resulting in the formation of insoluble aggregates causing turbidity. The autoanalyzer monitors the change in absorbance at 940 nm. This change in absorbance is proportional to the concentration of C-reactive protein in the sample. The dynamic range of the assay is 0.280 mg/L, with a limit of detection of 0.2 mg/L. At a level of 1.75 mg/L, the interassay coefficient of variation is 2.9% (*N* = 9).

#### 2.3.3. Ferritin

The ferritin assay was obtained from Dialab, Vienna, Austria. (nr.A01551). The assay of ferritin is based on turbidimetric measurement. Turbidity is caused by the formation of an insoluble immunocomplex of ferritin with a highly specific antibody. The dynamic range of the assay is 10–300 ng/mL, with a limit of detection of 10 ng/mL. At a level of 240 ng/mL, the interassay coefficient of variation is 2.4% (*N* = 9). Ferritin could not be measured in samples with a high lipemic index (*N* = 62, 31%).

#### 2.3.4. Transferrin

The kits for transferrin were purchased from Beckman Coulter Diagnostics (Woerden, The Netherlands). The transferrin assay is based on a turbidimetric method. In the reaction, transferrin combines with a highly specific antibody to form an insoluble antigen-antibody complex. The sensitivity for transferrin assay is 0.7 g/L, defined as the lowest measurable concentration which can be distinguished from zero with 95% confidence. At a level of 3.2 g/L, the interassay coefficient of variation is 1.2% (*N* = 9).

#### 2.3.5. Total Serum Iron

The kits for total serum iron were purchased from Beckman Coulter Diagnostics (Woerden, The Netherlands). The total iron assay is a colorimetric assay. The iron reagents are used to measure the iron concentration by a timed-endpoint method. In the reaction, iron is released from transferrin by acetic acid and is reduced to the ferrous state by hydroxylamine and thioglycolate. The ferrous ion is immediately complexed with the FerroZine Iron Reagent (3-2-pyridyl-5,6-diphenyl-1,2,4-triazine-p,p′-disulfonic acid monosodium salt hydrate). The sensitivity for the iron assy is 0.9 μmol/L, defined as the lowest measurable concentration, which can be distinguished from zero with 95% confidence. At a level of 28 μmol/L, the interassay coefficient of variation is 2.5% (*N* = 9). Total serum iron concentration was measured after acidifying the serum sample in order to release the iron bound to transferrin. 

#### 2.3.6. Transferrin Saturation

The transferrin saturation (TS%) was calculated by the equation shown below [31].

TS% = (Total serum iron [µmol/L]/Transferrin [mg/dL]) × 398(1)

### 2.4. Statistical Analyses

The characteristics of cases and controls were expressed as proportions or medians (interquartile ranges) for categorical and continuous variables, respectively. Furthermore, the characteristics of overall CRA and advanced CRA cases were compared with those of the controls by Wilcoxon rank-sum tests (for continuous variables) or chi-squared tests (for categorical variables). In addition, Kruskal–Wallis tests were applied to assess differences in biomarker distributions among subjects with low, intermediate and high red/processed meat consumption and BMI. Mutual correlations among SHP, hs-CRP, ferritin, transferrin, total serum iron and transferrin saturation levels were estimated by Spearman correlation coefficients.

Logistic regression, by which odds ratios (ORs) and 95% confidence intervals (CIs) were estimated, was applied to assess the associations of the serological biomarkers with overall and advanced CRA. Two multivariable models were applied. Model 1 was adjusted for age, sex, BMI, physical activity, smoking status, red meat and processed meat consumption. Model 2 was additionally adjusted for SHP, hs-CRP, ferritin and transferrin saturation levels. 

In sensitivity analyses, eight study participants with hsCRP levels > 10 mg/L were excluded because these levels could indicate an acute infection (of which three were adenoma cases). However, the results were not relevantly altered (data not shown). 

All statistical tests were two-sided using a significance level of 0.05. All analyses were performed with the Statistical Analysis System (SAS) version 9.4 (SAS Institute, Inc., Cary, NC, USA). Missing data were assumed to be missing at random conditional on observed data, and five datasets were imputed with multiple imputations by the SAS procedure PROC MI. All analyses were performed with the imputed datasets with the SAS procedure PROC MI-ANALYZE.

## 3. Results

Demographic characteristics and serological biomarkers distributions of the control participants, overall CRA and advanced CRA cases are shown in Table 1. For the comparison between CRA cases and controls, the age and sex distributions were the same because of the frequency-matching. Furthermore, no statistically significant difference was observed for BMI, smoking status, physical activity, red and processed meat consumption as well as for the levels of hs-CRP and ferritin. However, all CRA cases and the advanced CRA cases had significantly higher transferrin saturation levels than the controls. In addition, SHP levels were statistically significantly lower among advanced CRA cases than in controls.

Median ferritin levels stepwise increased from low and intermediate to high red and processed meat consumption and the Kruskal–Wallis test detected a statistically significant difference (Table 2). Although not statistically significant, the same trend was observed for hs-CRP levels. No associations with red and processed meat consumption were observed for the other biomarkers. 

SHP levels were significantly, inversely associated with the BMI. SHP levels were particularly low among subjects with BMI ≥ 30 kg/m^2^ (Table 3). Hs-CRP levels were significantly, positively associated with the BMI and median hs-CRP levels of obese subjects with BMI ≥ 30 kg/m^2^ were more than 4-fold higher than among lean individuals with BMI < 25 kg/m^2^. The biomarkers of body iron storage were not associated with obesity.

Spearman correlation coefficients (r) were estimated to assess correlations of SHP, hs-CRP, ferritin, transferrin, total serum iron and transferrin saturation levels with each other (Table 4). SHP was statistically significantly, positively correlated with transferrin (*r* = 0.22) and total serum iron (*r* = 0.223). The hs-CRP levels were statistically significantly, inversely associated with transferrin saturation (*r* = −0.19). Among the biomarkers for body iron storage, ferritin concentration was statistically significantly associated with transferrin levels (inversely, *r* = −0.19) and transferrin saturation (*r* = 0.18). Furthermore, transferrin levels, total serum iron levels and transferrin saturation were correlated with each other as expected.

Table 5 presents the association of serological biomarkers levels with overall CRA and advanced CRA in two models. Model 1 was adjusted for age, sex and lifestyle factors whereas model 2 additionally adjusted for SHP, hs-CRP, ferritin and transferrin saturation levels. As results for the two models did not differ much, we focus on the results from model 2. SHP levels were statistically significantly, inversely associated with advanced CRA (top vs. bottom tertile: OR [95% CI] = 0.29 [0.10–0.84]) but the association with overall CRA was not statistically significant (top vs. bottom tertile: OR [95% CI] = 0.65 [0.27–1.56]). Moreover, transferrin saturation was statistically significantly, positively associated with overall CRA (top vs. bottom tertile: OR [95% CI] = 3.05 [1.30–7.19]) and advanced CRA (top vs. bottom tertile: OR [95% CI] = 2.71 [1.03–7.13]). Because of the high correlation of total serum iron levels and transferrin saturation (*r* = 0.86), results for total serum iron levels were similar to those of transferrin saturation. However, effect estimates were a little weaker and not statistically significant. Furthermore, results for hs-CRP, ferritin and transferrin levels were not statistically significant.

In sensitivity analysis, the variables for the BMI, red meat and processed meat consumption were removed from the models but the results did not change to any relevant extent (data not shown). This can be explained by the circumstance that these variables were neither significantly associated with overall CRA nor advance CRA in this study (data not shown). Furthermore, no relevant differences were observed for analyses of Table 2, Table 3 and Table 4 when they were conducted stratified by adenoma case status (data not shown).

## 4. Discussion

In this cross-sectional analysis from a colonoscopy screening cohort, we investigated associations of serum biomarkers for body iron stores, inflammation, and oxidative stress with each other, with red and processed meat consumption and obesity as well as with overall and advanced CRA. Ferritin levels were significantly, positively associated with red and processed meat consumption and hs-CRP levels with obesity. SHP levels were significantly inversely associated with obesity and advanced CRA. Transferrin saturation and total serum iron levels were strongly, positively associated with overall and advanced CRA but only the results for transferrin saturation were statistically significant. The other biomarkers for body iron stores (ferritin and transferrin) and the biomarker for inflammation, hs-CRP, were not associated with CRA.

To the best of our knowledge, no single study has previously simultaneously investigated the associations of biomarkers for body iron stores, inflammation, and oxidative stress with CRA. However, previous studies, separately exploring the abovementioned biomarkers and CRA risk, have been conducted. 

Regarding hs-CRP, a meta-analysis with ten studies including a total of 3350 cases and 4168 controls observed no association of CRP levels with overall CRA, but a significant positive association of CRP concentration with advanced CRA [32]. In our study, hs-CRP levels were neither associated with overall nor advanced CRA. However, the confidence intervals were wide and low statistical power may have been the reason for not detecting the association of hs-CRP levels and advanced adenoma.

With respect to oxidative stress biomarkers, epidemiological studies observed that F2-isoprostanes, fluorescent oxidation products and 8-Oxo-2’-deoxyguanosine concentrations were positively associated with overall CRA risk [33,34,35]. Our study was the first to assess the association of SHP levels with CRA and observed a plausible inverse direction of the association of SHP levels with advanced CRA. The direction is plausible because SHP is a stable biomarker for the overall antioxidant capacity. Our study further observed that SHP levels were strongly decreased in obese study participants, which is in agreement with the results of a previous, longitudinal study from the general population [36]. Obesity is an established risk factor for CRA and CRC and there is evidence that the pathway is mainly via subclinical inflammation and oxidative stress [5,6,7,8]. White adipose tissue is regarded as an endocrine organ that produces pro-inflammatory cytokines like TNF-α, IL-1, and IL-6 [37]. These, in turn, increase the generation of ROS and subsequently decrease anti-oxidant capacities [38]. In addition, the gastrointestinal hormone ghrelin, which among other functions regulates appetite [39], has decreased serum levels in subjects with obesity and plays an important role in anti-inflammatory and anti-oxidative processes in the body [40,41]. Low serum levels of ghrelin have further been found to be associated with an increased risk of the development of CRC [42] and experimental studies with mice have shown that ghrelin administration can suppress inflammation-induced colorectal carcinogenesis [43,44,45]. Thus, the mechanisms by which obesity can cause CRC are quite well understood and a decreased antioxidant capacity is one of these mechanisms. The latter is well measured by SHP and thus this biomarker might be suitable to monitor the pathology from obesity to CRA. This is because the SHP assay is easy to measure at low costs with auto-analyzers.

In addition, biomarkers of lipid peroxidation may be useful in CRC risk assessment. In particular, 4-hydroxynonenal (HNE) and acrolein have been shown to be associated with CRC and other cancers [46,47,48,49,50]. Acrolein is highly reactive towards protein and DNA and was found to be associated with the transition from benign to colon tumors [49,50]. However, it has yet to be evaluated if acrolein has some physiological role despite being a carcinogen [50]. In contrast, HNE is involved in multiple signaling pathways and its actions are complex and can involve both cancer growth at low to moderate concentration as well as apoptosis of cancer cells at high concentration [47]. Other reduction–oxidation pathways involved in cancer development have been systematically reviewed elsewhere [51]. Both 4-HNE and acrolein are being removed from cells by glutathione [50], which supports the observed association of low SHP levels and advanced CRA.

Regarding biomarkers of body iron storage, to the best of our knowledge, associations between serum transferrin levels and risk of CRA have not been investigated by previous studies. Only four previous observational studies investigated the association of other serological iron biomarkers with CRA and their results were pooled by a meta-analysis, in which ferritin, iron and transferrin saturation were not associated with CRA [27]. The meta-analysis for transferrin saturation only included the prospective study by Cross et al. [25] and the cross-sectional study by Chan et al. [24]. In contrast to these two studies, our cross-sectional study observed a strong, positive association for transferrin saturation. On the first view, it is surprising that on the one hand, ferritin levels and not transferrin saturation were associated with red meat consumption and on the other hand transferrin saturation but not ferritin was associated with CRA. An explanation for these seemingly divergent findings may be that ferritin is the best biomarker to measure iron uptake from diet and the total body iron stores [22,38]. In contrast, transferrin saturation may be the better biomarker for tissue iron stores and may better detect iron overload. The picture becomes more clear when looking at experimental studies which have shown that dietary iron does not induce iron overload [52]. Thus, the observed association of transferrin saturation with CRA is likely a sign of iron overload that is not caused by excessive dietary haem consumption but by other reasons. One cause could be untreated hereditary hemochromatosis, which is rare but not extremely rare. In an analysis of the large UK Biobank study (*N* = 451,243), 0.6% of the study participants were homozygous for the genetic variant responsible for most hereditary haemochromatosis [53]. Thus, in our study population with *N* = 200, at least one case of hereditary haemochromatosis can be expected. If hereditary haemochromatosis is more frequent among individuals with CRA (which is unknown), or simply by chance, a few more cases of hereditary haemochromatosis could be among our CRA cases than would be expected by chance. However, this is just speculation and other studies are needed to assess whether people with hereditary haemochromatosis have a higher CRA risk. 

Although the reasons for the iron overload in our study remain largely unknown, the mechanism by which it could be related to CRA is known. Several studies have reported that iron overload mediated colonic tumorigenesis via increased ROS generation and inflammation [54]. One of the possible explanations is the synergistic effect of iron and non-amidated gastrins, including Gamide, progastrin and Ggly, which are peptide hormones regulating gastric acid secretion and gastric cell proliferation [54]. Non-amidated gastrins have been proposed to catalyze the loading of transferrin with iron [55]. There is also evidence that transferrin saturation and circulating gastrin levels are positively correlated in mice and humans [56]. Furthermore, it has been observed that plasma levels of progastrin are elevated in CRC patients, suggesting that non-amidated gastrins may accelerate the development of colorectal carcinoma [57]. Nevertheless, precise mechanisms underlying the involvement of non-amidated gastrins and iron overload are still not clear.

The limitations of the present study include the small sample size, the cross-sectional design and its observational nature. Therefore, causality cannot be determined and residual confounding cannot be completely excluded in this type of study. However, in order to control for confounding, cases and controls were frequency-matched by age and sex, and models were comprehensively adjusted for other potential confounders. Strengths of the study include the sampling of cases and controls from the same cohort of screening colonoscopy participants and the measurement of a large number of biomarkers. 

## 5. Conclusions

The current study observed that the levels of the oxidative stress biomarker SHP were inversely associated with the BMI and advanced CRA, suggesting that low SHP levels may reflect one possible mechanism linking obesity with CRA risk. Furthermore, transferrin saturation was positively associated with overall and advanced CRA risk. This finding could not be explained by red and processed meat consumption and is likely rather a result of iron overload among CRA cases that had other causes (maybe hereditary hemochromatosis). Nevertheless, transferrin saturation might be a useful biomarker for identifying patients with iron overload, which is also a risk factor for CRA. The potential of SHP levels and transferrin saturation for risk stratification in CRC screening should be followed up in further, much larger studies.

## Figures and Tables

**Table 1:** Baseline characteristics of adenoma case patients as well as age and sex frequency-matched control participants.

Characteristics	Controls			Overall CRA Cases			Advanced CRA Cases		
	*n*	Median (IQR)	%	*n*	Median (IQR)	%	*n*	Median (IQR)	%
**Age (years)**	100	60 (57–67)	-	100	60 (57–67)	-	71	60 (56–67)	-
**Sex**									
Female	55	-	55.0	55	-	55.0	43	-	60.6
Male	45	-	45.0	45	-	45.0	28	-	39.4
**BMI (kg/m^2^)**									
<25	34	-	34.7	31	-	32.3	26	-	38.8
25–< 30	46	-	46.9	48	-	50.0	30	-	44.8
≥30	18	-	18.4	17	-	17.7	11	-	16.4
**Smoking status**									
Never smoker	58	-	58.6	42	-	42.4	30	-	42.3
Former smoker	33	-	33.3	45	-	45.5	31	-	43.7
Current smoker	8	-	8.1	12	-	12.1	10	-	14.1
**Physical activity**									
Inactive	44	-	47.3	42	-	45.7	31	-	47.7
Sedentary	33	-	35.5	28	-	30.4	23	-	35.4
Vigorously	16	-	17.2	22	-	23.9	11	-	16.9
**Red and processed** **meat consumption ^a^**									
Low	12	-	12.2	14	-	14.0	12	-	16.9
Intermediate	70	-	71.4	80	-	80.0	54	-	76.1
High	16	-	16.3	6	-	6.0	5	-	7.0
**SHP (μmol/L)**	100	258 (205–290)	-	100	228 (200–275)	-	71	**218 (188–265)**	-
**hs-CRP (mg/L)**	91	1.44 (0.59–3.26)	-	97	1.21 (0.64–1.99)	-	70	1.16 (0.54–1.96)	-
**Ferritin (ng/mL)**	68	86.2 (45.6–190.4)	-	70	78.7 (52.4–140.9)	-	51	73.4 (52.4–133.9)	-
**Transferrin (mg/dL)**	97	224 (207–251)	-	96	229 (205–262)	-	67	221 (203–253)	-
**Total serum iron (μmol/L)**	99	13.9 (11.0–18.1)	-	100	15.2 (11.3–19.7)	-	71	15.2 (11.0–19.5)	-
**Transferrin saturation (%)**	97	23.9 (18.8–30.1)	-	96	**26.7 (21.6–34.7)**	-	67	**27.2 (21.6–35.2)**	-

Abbreviations: BMI, body mass index; hs-CRP, high-sensitive C-reactive protein; IQR, interquartile range; SHP, total thiol levels. Note: Numbers printed in bold: Statistically significant difference of controls with overall CRA cases and advanced CRA cases (*p* < 0.05; chi-squared test for categorical variables and Wilcoxon rank-sum test for continuous variables). ^a^ Low red and processed meat consumption was defined as both red and processed meat consumption no more than once per week. High red and processed meat consumption was defined as both daily red and processed meat consumption or either red or processed meat consumption several times per day. All others were grouped in the intermediate consumption category.

**Table 2:** Differences in median concentrations of biomarkers of oxidative stress, inflammation and body-iron stores according to red and processed meat consumption.

Biomarker	Red and Processed Meat Consumption ^a^			
	Low	Intermediate	High	*p* Value ^b^
	**(*N* = 26)**	**(*N* = 150)**	**(*N* = 22)**	
	**Median (IQR)**	**Median (IQR)**	**Median (IQR)**	
SHP (μmol/L)	254 (211–306)	239 (200–281)	246 (215–285)	0.419
hs-CRP (mg/L)	0.94 (0.51–2.12)	1.30 (0.65–2.37)	1.71 (0.81–2.06)	0.340
Ferritin (ng/mL)	**54.8 (42.3–84.5)**	**84.0 (52.4–160.6)**	**158.5 (99.0–226.9)**	**0.006**
Transferrin (mg/dL)	240 (219–263)	222 (202–253)	228 (215–240)	0.162
Total serum iron (μmol/L)	16.4 (13.4–19.0)	14.1 (11.0–18.2)	15.0 (12.2–20.2)	0.308
Transferrin saturation (%)	25.6 (20.1–32.4)	24.5 (20.1–33.1)	28.8 (21.3–34.8)	0.546

Abbreviations: hs-CRP, high-sensitive C-reactive protein; IQR, interquartile range; SHP, total thiol levels. ^a^ Low red and processed meat consumption was defined as both red and processed meat consumption no more than once per week. High red and processed meat consumption was defined as both daily red and processed meat consumption or either red or processed meat consumption several times per day. All others were grouped in the intermediate consumption category. ^b^ Kruskal–Wallis test. Notes: Bold print indicates statistically significant differences (*p* < 0.05). No relevant differences were observed when the analysis was conducted stratified by adenoma case status (data not shown).

**Table 3:** Differences in median concentrations of biomarkers of oxidative stress, inflammation and body-iron stores according to body mass index.

Biomarker	Body Mass Index			
	<25 kg/m^2^	25–<30 kg/m^2^	≥30 kg/m^2^	*p* Value ^a^
	**(*N* = 65)**	**(*N* = 94)**	**(*N* = 35)**	
	**Median (IQR)**	**Median (IQR)**	**Median (IQR)**	
SHP (μmol/L)	**258 (206–285)**	**251 (207–287)**	**215 (163–266)**	**0.044**
hs-CRP (mg/L)	**0.87 (0.41–1.81)**	**1.40 (0.78–2.14)**	**3.69 (1.10–5.54)**	**<0.001**
Ferritin (ng/mL)	84.1 (51.0–140.9)	76.7 (48.1–154.2)	108.6 (45.2–226.9)	0.601
Transferrin (mg/dL)	224 (212–251)	228 (201–253)	226 (212–264)	0.501
Total serum iron (μmol/L)	15.2 (12.2–19.0)	14.3 (11.3–18.7)	12.9 (9.8–17.6)	0.246
Transferrin saturation (%)	25.6 (21.6–34.1)	25.6 (21.3–34.7)	20.6 (17.1–29.3)	0.087

Abbreviations: hs-CRP, high-sensitive C-reactive protein; IQR, interquartile range; SHP, total thiol levels. ^a^ Kruskal–Wallis test. Notes: Bold print indicates statistically significant differences (*p* < 0.05). No relevant differences were observed when the analysis was conducted stratified by adenoma case status (data not shown).

**Table 4:** Correlation matrix for biomarkers of oxidative stress, inflammation and body-iron stores.

	SHP	Hs-CRP	Ferritin	Transferrin	Total Serum Iron	TS %
SHP	-	*r* = −0.067 *p* = 0.359 *N* = 188	*r* = −0.041 *p* = 0.634 *N* = 138	***r*****= 0.216** ***p*****= 0.003** *N* = 193	***r*****= 0.223** ***p*****= 0.002** *N* = 199	*r* = 0.082 *p* = 0.257 *N* = 193
Hs-CRP	-	-	*r* = 0.078 *p* = 0.374 *N* = 131	*r* = 0.053 *p* = 0.475 *N* = 184	*r* = −0.066 *p* = 0.367 *N* = 188	***r*****= −0.191** ***p*****= 0.009** *N* = 184
Ferritin	-	-	-	***r*****= −0.186** ***p*****= 0.031** *N* = 134	*r* = 0.075 *p* = 0.380 *N* = 138	***r*****= 0.175** ***p*****= 0.043** *N* = 134
Transferrin	-	-	-	-	***r*****= 0.266** ***p*****< 0.001** *N* = 193	***r*****= −0.180** ***p*****= 0.013** *N* = 193
Total serum iron	-	-	-	-	-	***r*****= 0.861** ***p*****< 0.001** *N* = 193

Abbreviations: hs-CRP, high sensitive C-reactive protein; r, Spearman correlation coefficient; TS %, transferrin saturation percentage; SHP, total thiol levels. Notes: Bold print indicates statistically significant correlation. No relevant differences were observed when the analysis was conducted stratified by adenoma case status (data not shown).

**Table 5:** Odds ratios and 95% confidence intervals for the associations of biomarkers of oxidative stress, inflammation and body-iron stores concentration tertiles with overall as well as advanced colorectal adenoma.

Biomarker	Categories	Concentrations	*n*_control_	Overall Colorectal Adenoma			Advanced Colorectal Adenoma		
				*n*_case_	Model 1 OR (95%CI) ^a^	Model 2 OR (95%CI) ^b^	*n*_case_	Model 1 OR (95%CI) ^a^	Model 2 OR (95%CI) ^b^
**SHP (μmol/L)**	**Tertile 1**	**≤216**	34	40	Ref.	Ref.	33	Ref.	Ref.
	Tertile 2	>216–≤280	33	37	1.07 (0.52–2.22)	0.86 (0.40–1.87)	25	0.76 (0.34–1.68)	0.58 (0.25–1.40)
	Tertile 3	>280	33	23	0.73 (0.32–1.69)	0.65 (0.27–1.56)	13	**0.32 (0.12–0.89)**	**0.29 (0.10–0.84)**
**hs-CRP (mg/L)**	Tertile 1	≤0.83	30	33	Ref.	Ref.	26	Ref.	Ref.
	Tertile 2	>0.83–≤2.02	30	41	1.22 (0.58–2.54)	1.37 (0.65–2.92)	29	1.12 (0.50–2.52)	1.50 (0.63–3.56)
	Tertile 3	>2.02	31	23	0.65 (0.28–1.51)	0.77 (0.32–1.91)	15	0.46 (0.17–1.24)	0.64 (0.22–1.87)
**Ferritin (ng/mL)**	Tertile 1	≤56.2	23	21	Ref.	Ref.	15	Ref.	Ref.
	Tertile 2	>56.2–≤138.6	23	31	1.36 (0.55–3.31)	1.81 (0.74–4.39)	25	1.86 (0.67–5.13)	1.87 (0.67–5.25)
	Tertile 3	>138.6	22	18	0.88 (0.32–2.41)	0.93 (0.32–2.74)	11	0.77 (0.24–2.49)	0.72 (0.21–2.51)
**Transferrin (mg/dL)**	Tertile 1	≤212	33	29	Ref.	Ref.	22	Ref.	Ref.
	Tertile 2	>212–≤241	33	27	1.26 (0.57–2.78)	0.97 (0.44–2.16)	20	1.07 (0.45–2.54)	0.79 (0.32–1.96)
	Tertile 3	>241	32	40	1.45 (0.68–3.10)	1.14 (0.51–2.50)	25	1.03 (0.44–2.42)	0.84 (0.34–2.05)
**Total serum iron** **(μmol/L)**	Tertile 1	≤11.9	33	28	Ref.	Ref.	21	Ref.	Ref.
	Tertile 2	>11.9–≤15.9	33	27	1.12 (0.50–2.49)	1.07 (0.47–2.45)	19	0.94 (0.38–2.28)	0.99 (0.39–2.53)
	Tertile 3	>15.9	33	45	2.04 (0.95–4.39)	2.07 (0.91–4.70)	31	1.63 (0.71–3.75)	2.10 (0.82–5.40)
**Transferrin** **saturation (%)**	Tertile 1	≤20.7	33	19	Ref.	Ref.	15	Ref.	Ref.
	Tertile 2	>20.7–≤27.4	32	31	2.24 (0.95–5.28)	2.07 (0.86–5.01)	19	1.56 (0.59–4.10)	1.56 (0.54–4.48)
	Tertile 3	>27.4	32	46	**3.98 (1.70–9.32)**	**3.05 (1.30–7.19)**	33	**3.48 (1.38–8.75)**	**2.71 (1.03–7.13)**

Note: Numbers in bold reflect the statistically significant estimates compared to the reference (*p* < 0.05). Abbreviations: CRA, colorectal adenoma; Carr U, Caratelli units; hs-CRP, high sensitive C-reactive protein; SHP, total thiol levels. ^a^ The logistic regression model is adjusted for age, sex, BMI, physical activity, smoking status, red and processed meat consumption. ^b^ The logistic regression model is adjusted for variables of model 1, SHP, hs-CRP, ferritin and transferrin saturation (except for analyses with transferrin and total serum iron, which were not adjusted for transferrin saturation because they are being used to calculate transferrin saturation).

## Data Availability

The data presented in this study are available on request from the corresponding author. The data are not publicly available due to privacy restrictions.
